# Embedding collective leadership to foster collaborative inter-professional working in the care of older people (ECLECTIC): Study protocol

**DOI:** 10.12688/hrbopenres.13004.1

**Published:** 2020-03-03

**Authors:** Sabrina G. Anjara, Éidín Ní Shé, Marie O'Shea, Gráinne O'Donoghue, Sarah Donnelly, John Brennan, Hellen Whitty, Paul Maloney, Anne Claffey, Siobhan Quinn, Niamh McMahon, Noeleen Bourke, Deirdre Lang, Patrice Reilly, Catherine McGuigan, Sarah Cosgrave, Louise Lawlor, Diarmuid O'Shea, Eilish McAuliffe, Deirdre O'Donnell

**Affiliations:** 1 School of Nursing, Midwifery and Health Systems, University College Dublin, Belfield, Dublin, 4, Ireland; 2 School of Public Health, Physiotherapy and Sports Science, University College Dublin, Belfield, Dublin, 4, Ireland; 3 School of Social Policy, Social Work and Social Justice, University College Dublin, Belfield, Dublin, 4, Ireland; 4 National Clinical Programme for Older People, Royal College of Physicians of Ireland, Dublin, 2, Ireland; 5 Beaumont Hospital, Dublin, 9, Ireland; 6 Regional Hospital Mullingar, Mullingar, N91 NA43, Ireland; 7 Tallaght University Hospital, Dublin, 24, Ireland; 8 St. James’s University Hospital, Dublin, 8, Ireland; 9 School of Pharmacy and Pharmaceutical Sciences, Trinity College Dublin, Dublin, 2, Ireland; 10 Health Service Executive CHO 8 (Longford and Westmeath), Mullingar, Ireland; 11 Health Service Executive, Dublin, 8, Ireland; 12 Health Service Executive CHO 9 (Dublin North City and County), Dublin, Ireland; 13 Age Friendly Ireland, Navan, Ireland; 14 St. Vincent’s University Hospital, Dublin, 4, Ireland

**Keywords:** Co-design, Integrated Care, Delivery of Health Care, Older Adults, Collective Leadership

## Abstract

**Background: **The National Integrated Care Programme for Older People (NICPOP), formerly NCPOP aims to support older people to live well in their homes by developing primary and secondary care services for older people, especially those with complex needs. The programme develops integrated intermediate care which traverses both hospital and community settings through multidisciplinary and interagency teams. This team-based approach to the integration of health services is a novel innovation in Irish health service delivery and will require, over time, a shift in cultures of care to allow for the development of competencies for inter-professional collaboration across the care continuum.

The ECLECTIC project will develop an implementation framework for achieving, maintaining and monitoring competencies for interprofessional collaboration among multi-disciplinary teams charged with delivering care for older people across the continuum from acute to community settings.

**Design: **The ECLECTIC research design has been developed in collaboration with the NICPOP. In phase one of the project, a co-design team will collaborate to define and shape competencies for interprofessional collaboration. Phase two will involve the delivery of a collective leadership intervention over a 10-month period with multidisciplinary professionals working with older people across two geographical regions (Mullingar/Midlands and Beaumont/Dublin North). Each group will comprise of members of two multidisciplinary teams charged with coordinating and delivering care to older people across the continuum of acute to community care. Observations of collaborative inter-professional working will take place before, during, and after intervention. In phase three of the study, analysis of the interview and observation data will be presented to the co-design team in order to develop an implementation framework for future teams.

**Discussion:** The co-design process will develop core competencies and performance indicators for collaborative interprofessional working. The resulting implementation framework will be implemented nationally as part of the NICPOP.

## Introduction

Over the next 30 years the number of people in Ireland aged 65 and older is projected to increase by 60–64% and the numbers aged 85 and older by 89–94% ( Ní Shé *et al.*, 2018b). The health system will, therefore, have to respond to this changing demographic and their specific healthcare requirements ( Ireland, 2017; McLoughlin, 2014; Office, 2013; O’Donnell *et al.*, 2019). The National Integrated Care Programme for Older Persons (NICPOP) aligns the programmes of work of the National Clinical Programme for Older People (NCPOP) and the Integrated Care Programme for Older People (ICPOP) under the leadership of Dr Siobhán Kennelly (National Clinical Advisor and Group Lead – HSE). It is a joint initiative between the Clinical Strategy and Programmes Division of the Health Service Executive (HSE) and the Royal College of Physicians of Ireland (RCPI) (Executive). The aim of the NICPOP is to improve and standardise the quality of health and social care for older people in Ireland by supporting the provision of services across all settings. The necessity to develop and support interprofessional collaboration in the case management of older people’s care has been identified as a key enabler for improving health and social care delivery. As such, the Inter-Professional Interest Group, a sub-committee of the NICPOP, was established to inform the strategic development of improved collaborative interprofessional working across the boundaries of acute to community health and social care with respect to older people.

Integrated Care (IC) has been adopted in Irish health and social care policy as a strategy for improving the coordination of health and social services across the continuum from acute to community care ( Ní Shé *et al.*, 2018b). Research evidence indicates that models of IC may enhance patient satisfaction, increase perceived quality of care and, importantly in the context of increasing demand, enable improved access to services ( Baxter *et al.*, 2018). *SláinteCare* is the first Irish health and social care policy to receive cross-party commitment guaranteeing a 10-year implementation plan ( SláinteCare, 2017). The report orientates the health system towards universal access and proposes the development of a new model of coordinated health and social care shifting the focus of service delivery from acute to community. IC is central to this vision as it emphasises cross-agency and multidisciplinary case management and improves access to care in the community.

Aligned to the shifting of focus of older people’s care from the acute-centric model is the intention to establish integrated intermediate care which traverses the hospital and community in the form of multidisciplinary and interagency teams. A total of thirteen pioneer Integrated Care Teams (ICTs) were established within the nine HSE Community Healthcare Organisations (CHOs) which govern service delivery in local communities across the country. The implementation of the ICTs saw the establishment of new community roles for health and social care professionals to engage in a team-based case management approach to coordinating care of older people ( Executive, 2018). This team-based approach to the integration of health services is a novel innovation in Irish health service delivery and will require over time a shift in cultures of care to allow for the development of competencies to foster inter-professional collaboration across the care continuum.

*Our working definition of ICTs follow the European Competency Framework for Health and Social Care Professionals ( Dijkman *et al.*, 2016) and includes all professions working along the continuum of care for older people. In the Irish context, this can include inter alia people working within Emergency Departments’ Frailty Intervention Therapy Teams, Community Health Organisations, Primary Care Teams, Safeguarding and Protection Teams, Mental Health Teams. These teams encompass all health and social care disciplines including nursing and pharmacy.*

The European Competency Framework for Health and Social Care Professionals (HSCPs) working with older people ( Dijkman *et al.*, 2016) describes the outcomes that HSCPs are expected to achieve and demonstrate in their different roles (7 roles in total). The framework describes a minimum set of competencies (18 in total) that constitute a common baseline for HSCPs working with older people and their families within their local communities ( Dijkman *et al.*, 2016). Each of the role-specific competencies is presented with performance indicators that are skills, behaviours or practices that demonstrate competence. This framework, alongside the Irish educational framework for gerontological nursing ( Coffey *et al.*, 2016) will provide a foundation for ECLECTIC in building an understanding of core competencies for inter-professional collaboration across HSCPs. These competencies will be aligned to indicators of appropriate skills, behaviours and practices fostering collaborative working and collective leadership within multidisciplinary teams charged with integrating health and social care for older people in their communities.

While a strategic emphasis on IC is currently at the centre of the Irish health and social care policy, clarity is required regarding the aims of integration, what services are included, which professionals should be involved and how interagency collaboration is occurring ( Cameron *et al.*, 2014; Shaw *et al.*, 2011). Internationally the literature points to cultural and structural barriers to inter-professional collaboration across the continuum of care ( Ahmed *et al.*, 2015; Ní Shé *et al.*, 2018b). More specifically, there is a knowledge gap concerning how different health and social care professionals can foster new ways of collaborative working which are foundational to the implementation of IC cultures ( Ahmed *et al.*, 2015; Cameron *et al.*, 2014). At the centre of the drive for greater integration of services is inter-professional collaboration along a continuum of care ( Ní Shé *et al.*, 2018b; Van der Heide *et al.*, 2018). Therefore, if the policy agenda on integration is to be realised, it is necessary to understand the competencies and cultures that underpin collaborative approaches to care. This requires a reconceptualisation of leadership and roles within teams responsible for healthcare management and service delivery.

The collective approach to leadership has been defined as a dynamic team phenomenon, where leadership roles are distributed and shared among the team ( D’Innocenzo *et al.*, 2016). This approach requires individuals to adopt leadership roles where they have the expertise and motivation ( Friedrich *et al.*, 2009). Collective and shared approaches to leadership have been found to enhance team effectiveness and team performance outcomes ( D’Innocenzo *et al.*, 2016; Wang *et al.*, 2014). There is now considerable evidence for the effectiveness of collective leadership interventions in healthcare settings ( De Brún *et al.*, 2019). These studies have indicated the positive impact of collective leadership interventions on staff engagement, quality improvement, team-working and patient satisfaction ( De Brún *et al.*, 2019). Given this evidence base, there have been calls to move from traditional models to shared and distributed models of leadership in healthcare settings where increasingly, care is delivered via multidisciplinary teams ( West *et al.*, 2014; West *et al.*, 2015).

Effective teamwork is pertinent to inter-professional collaboration, especially for IC. There is limited guidance in the literature regarding how inter-professional collaboration could be fostered and sustained. Collective leadership, given its focus on harnessing collective intelligence towards collective action, and taking collective responsibility for shared impact, seems an appropriate mechanism by which multidisciplinary healthcare teams can demonstrate interprofessional collaboration through improving communication, team-working, person-centred care, and role clarity.

‘Collective Leadership and Safety Cultures’ (Co-Lead) is a 5-year mixed method research and capacity building programme led by Professor Eilish McAuliffe (co-author) ( McAuliffe *et al.*, 2017). The Co-Lead curriculum has been co-designed by healthcare staff, patient representatives and researchers ( Ward *et al.*, 2018). The programme involves a series of team-based training modules which aim to develop a dynamic leadership culture within multidisciplinary healthcare teams. Rather than starting from a top-down competency framework-driven curriculum targeted at the individual as leader, development is informed through a bottom-up service needs driven co-designed curriculum targeted at team members as co-leaders ( Ward *et al.*, 2018).

The ECLECTIC project will implement the Co-Lead intervention with multidisciplinary teams working with older people across acute and community care settings. The teams will be observed undertaking this intervention in order to gain insight into how multi-disciplinary teams collaborate when coordinating and delivering IC for older people. Six Co-Lead modules have been selected by the project team for implementation:

1. Team Values, Vision, and Mission
2. Team Goal Setting
3. Role Clarity
4. Collective Leadership for Safety Skills
5. Enhancing Person-centred Care
6. Monitoring and Communicating Safety Performance at Team Level

We hypothesise that these modules will enable teams to demonstrate the competencies for inter-professional collaboration, as determined by the co-design process.

### Study aim

We aim to collaborate with knowledge users, including public and patient representatives, in defining the core competencies for inter-professional collaborative working in health and social care teams providing services to older people.

The primary outcome of this project will be an implementation guide that details the core competencies for collaborative inter-professional working and provides a framework for achieving, maintaining and monitoring these competencies within multidisciplinary teams working across the continuum of IC for older people.

Specific objectives are:

1. To co-design competencies and performance indicators for inter-professional collaboration, building on the European Competency Framework for HSCPs working with Older People and the Irish educational framework for gerontological nursing.
2. To co-design with public and patient representatives (PPRs) two qualitative interview guides and two simulated scenarios/case studies. These will be used as data collection materials when observing ICT members’ approaches to patient care before, during and after the collective leadership interventions.
3. To implement six Co-Lead modules and evaluate their efficacy in fostering inter-professional collaboration among the ICTs.
4. To provide an implementation framework for achieving, monitoring and sustaining ways of collaborative inter-professional working which will be implemented nationally as part of the Integrated Care Programme for Older People.

## Methods

### Participants

The research acknowledges that by reason of the structure of health systems, individual health and social care professionals, particularly those who work in the hospital settings, may belong to more than one or even two teams. They will be part of their own disciplinary team, for example, physiotherapy, and they may also be a member of another team in an acute hospital service, for example, the Frail Intervention Therapy Team (FITT), and they may belong to a ward team. Each team has its own culture and ways of working, which will include how members communicate with one another. The term, “integrated care team” (ICT), in the context of ECLECTIC refers to the team of health and social care professionals who are involved in the care of older people across the hospital and community spectrum. These team members work together often, or from time-to-time and include those who discharge older people from hospital into a community service and vice versa as well as professionals based in the community who provide health and social care services to the older person.

### Procedure

**Figure 1.**
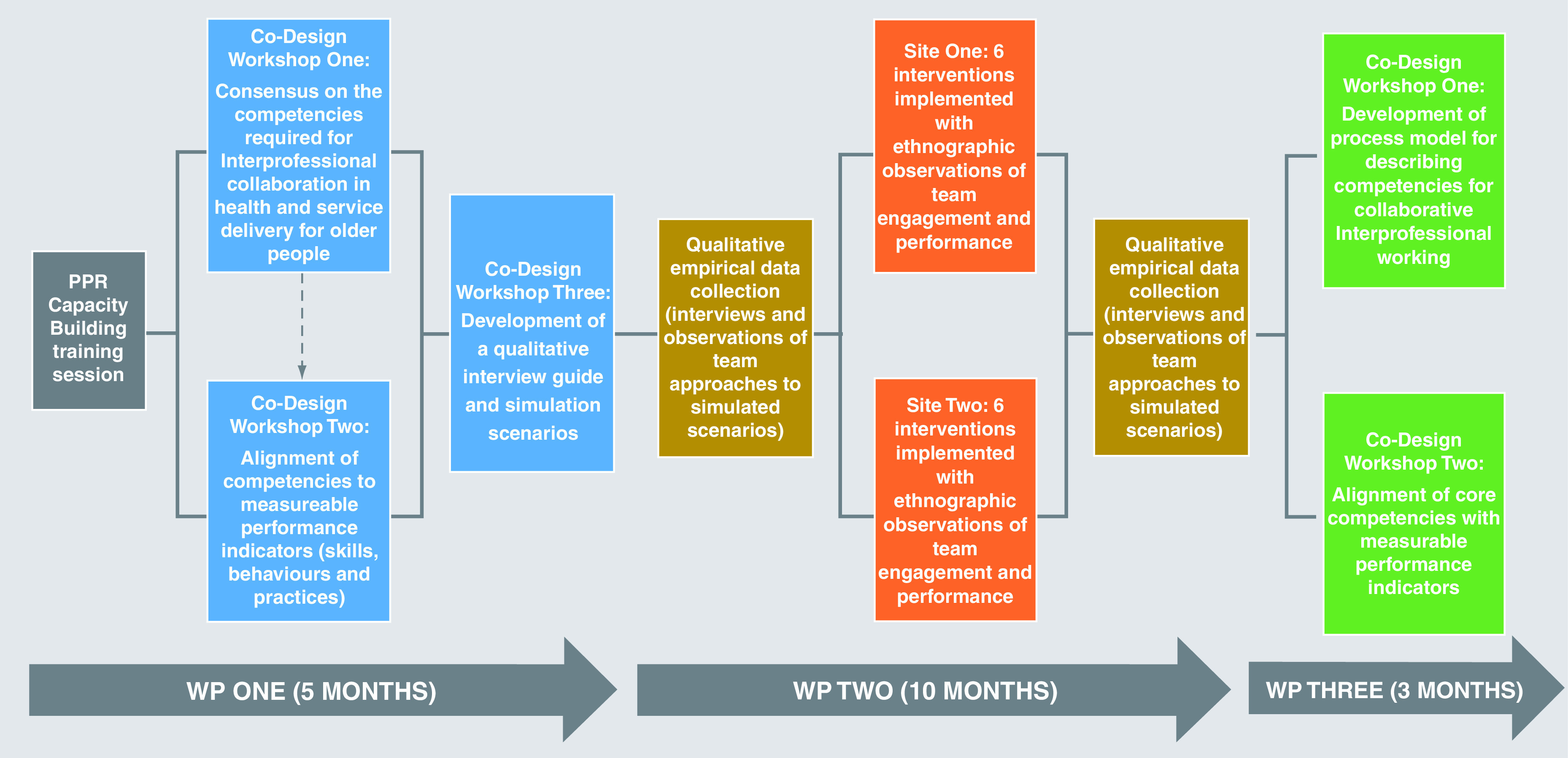
ECLECTIC Work Packages (WP) and timeline.

The ECLECTIC research design has been developed in collaboration with the Inter-professional Interest Group of the NICPOP. It is mapped out over three consecutive work packages ( Figure 1) combining a multi-method approach, including co-design workshops, outcome measurement development, qualitative data collection, intervention implementation and ethnographic observations.

***Work Package One*** commences with a capacity building training workshop for four PPRs nominated by Age Friendly Ireland who are collaborators for this proposal. This capacity training is essential in order to support democratic participation through a receptive research environment as well as clarifying roles and expectations regarding participation. The training will incorporate an understanding of the policy and service delivery context of IC for older people in Ireland including key terms, an outline of key policy documents as well as definitions and descriptions of the professionals involved. The session will also allow for clarification of the project objectives and governance. All PPRs will be adequately supported to participate including financial compensation for out of pocket costs as well as the establishment of a project link and PPR coordinator (ENS) who will communicate with the PPRs regularly as informal liaisons.

Subsequent to the capacity training, three co-design workshops will be held which will include PPRs, all co-applicants and collaborators for the ECLECTIC proposal. This will involve the health and social care disciplinary leads from the NICPOP Inter-Professional Interest Group, the National Clinical Lead for Older People (DO’S), the Director of Nursing for the NCPOP (DL) and the NCPOP programme manager (HW). The team will also include researcher co-authors from UCD and will be chaired by the PI (DO’D) who has extensive experience of meaningfully involving PPRs in co-designing healthcare interventions and services ( O'Donnell *et al.*, 2016; O’Donnell *et al.*, 2018; O’Donnell *et al.*, 2019).

A co-design approach within a health system improvement initiative involves creating an equal partnership of people working within the system and those individuals who have lived experience of using the system ( Ní Shé *et al.*, 2018a; O’Donnell *et al.*, 2018). We will follow the four pillars of effective and meaningful co-design with PPR involvement to ensure authentic and democratic participation and collaboration ( Baxter *et al.*, 2018; O'Donnell *et al.*, 2016; O’Donnell *et al.*, 2019). These pillars are research environment and receptive contexts; expectations and role clarity; support for participation and inclusive representation, as well as commitment to the value of co-learning involving institutional leadership.

The co-design workshops will build incrementally towards the development of consensus on descriptors of core competencies for inter-professional collaboration as well as performance indicators which will identify measurable skills, behaviours and practices. The European Competency Framework for HSCPs working with Older People ( Dijkman *et al.*, 2016) will provide the building material for the first co-design meeting, facilitating discussion of the characteristics of cohesive health care teams and the shaping of core competencies for collaboration. These competencies will be aligned with measurable outcomes and performance indicators (Objective One).

The second workshop will focus on identifying performance indicators of team cohesion, collective leadership and collaboration within and across healthcare settings relevant to the integration of care for older people. Anticipated output includes core measurable competencies for inter-professional collaborative working, identifying measurable outcomes and indicators of performance. These competencies will be mapped to the content and focus areas of the six Co-Lead modules.

In the third workshop, the co-design team will develop a qualitative interview guide and simulation scenarios for the purpose of generating qualitative observations of team approaches to patient care. The aim of the interview guide will be to prompt individual team members to explore and discuss their understandings of the necessary competencies for multidisciplinary and collaborative working and the performance indicators that they would recognise as being aligned to these competencies. Multidisciplinary simulation is a recognised pedagogical strategy for the development of clinical skills and the evaluation of team performance in the absence of patient risk ( Maxson *et al.*, 2011; Rodehorst *et al.*, 2005). A multidisciplinary approach to simulation has been used to successfully explore team roles and improve clinical case management ( Rodehorst *et al.*, 2005). The co-design team will develop simulation scenarios from their own practice experience which will be presented to the two ICTs before and after the collective leadership intervention. These scenarios will facilitate observation of the clinical case management approach of the ICTs in relation to competencies for inter-professional collaboration.

***Work Package Two*** aims to observe in vivo the competencies and performance indicators developed by the co-design process, within the real-world contexts of two multi-professional teams delivering care to older people across the acute and community setting. This will be achieved through implementing a collective leadership intervention over a 10-month period. Two study sites, rural and urban (Mullingar/Midlands and Beaumont/Dublin North), have been identified. Each team will be multidisciplinary in their composition, represent acute as well community care settings and will be working together to provide care for the same patients. Observing the teams before, during, and after intervention will allow for the comparison of contextual information pertaining to team cohesion and collaboration from an urban and rural location.

The sampling allows for comparisons between a pioneer ICT operating under NICPOP and a multi-disciplinary team who are operating across the boundary of community and acute care but who are not formally a part of the NICPOP. This will be important for the planned implementation of the ECLECTIC study findings within the NICPOP and more specifically for plans to develop further ICTs throughout the country. In order to champion this project within the two sites and to facilitate access to the relevant teams, representatives from the acute and community settings for both teams are included as members of the project team (AC, LL and PM – hospital representatives; NB and PR – community representatives).

Work Package Two will be a five-step process involving the gathering of qualitative empirical data through semi-structured interviews, observations of team approaches to simulated scenarios as well as ethnographic observations of the teams engaging with six Co-Lead modules (Objective Three). It will commence with semi-structured qualitative interviews of individual team members from both sites. Exploratory qualitative investigation is appropriate for this component of the project as little is known about how individual team members interpret and understand inter-professional collaboration in relation to their own disciplinary competency as well as in relation to the integration of patient centred care.

The semi-structured individual interviews will be complemented by observation of the team approach to simulated scenarios. Scenarios co-designed in the first work package will be presented to the teams and observations will be made of how the team approach these cases in terms of inter-professional collaboration. The observations will be structured and focused on identifying ‘competencies in action’, which demonstrate interprofessional team working as well as cross-agency collaboration. Analysis of the qualitative data generated from the interviews and observations will reveal contextual information in relation to how competencies for collaboration are operationalised and demonstrated within the two teams.

Following qualitative data collection (interviews and observations) the teams will undergo the collective leadership intervention. The six modules have been selected by the NICPOP and the Inter-Professional Interests Group from the 19 available as part of the Co-Lead programme. They will have been further mapped to the competencies for collaborative inter-professional working during the ECLECTIC co-design process. Each module is a one-hour team-based workshop designed to be self-directed by the team and implemented in their regular work environment.

The Co-Lead modules will have an emphasis on establishing the vision, mission and goals of an ICT as well as developing a series of team goals for achieving competency in inter-professional working and interagency collaboration. The modules will prompt team members to develop self-assessment criteria to monitor progress toward achieving these goals as well as maintaining and sustaining progress. The teams will be observed as they undertake each module and field notes will be taken which will focus on identifying ethnographic information regarding the culture of the team as well as contextual information pertaining to performance indicators for collaborative working.

Following completion of the six modules, qualitative exploration of the teams in relation to the operationalising of competencies for inter-professional collaboration will be repeated. Semi-structured interviews with each of the team members will be undertaken in order to explore how the collective leadership intervention have impacted upon their understanding of inter-professional collaboration and the integration of patient-centred care. The teams will be observed again in their approach to simulated scenarios that represent the complexities of integrating care for older people along a continuum from acute to community settings. The observations will focus on the operationalising of competencies for inter-professional collaboration in relation to simulated case management. The analysis of the qualitative data generated from this repeat wave of interviews and observations will evaluate the impact of the collective leadership intervention on an evolving team understanding of competencies for collaboration. Contextual information on how competencies are operationalised in terms of performance indicators and team cultures will be gathered to inform the implementation framework which will be the focus of the third work package.

***Work Package Three*** aims to develop an implementation framework for achieving, monitoring and sustaining ways of collaborative interagency working. This framework will be adopted by the NICPOP and used to support implementation at service delivery level. The framework will present the agreed core competencies for inter-professional collaborative working and align these competencies with measurable outcomes and performance indicators. There will be clear recommendations provided in the implementation strategy of the framework which will guide the NICPOP as to the application of the competencies and performance indicators within the national programme of ICTs for older people.

This work package will comprise of two co-design workshops over a three-month period. The co-design team from Work Package One will return for the workshops. In the first meeting, the co-design team will be presented with a synthesis of the qualitative data analysis from Work Package Two. The interview and observational data will be explored to describe current practice relating to inter-professional and interagency collaboration in the care of older people. This will involve identification of behaviour patterns (including leadership style), competency requirements, guidelines as well as situational or contextual influences which would facilitate achievement and sustainability of goals in relation to processes for inter-professional and interagency collaboration. The first co-design meeting will result in a working process model for describing competencies for collaborative inter-professional working within an ICT for older people.

A second co-design workshop will be focused on developing a framework for achieving, monitoring and maintaining new ways of collaborative and interagency working which emphasises the collective leadership capacity of the team (Objective Four). Discussions will focus on identifying processes for demonstrating and/or achieving competencies in relation to collaborative inter-professional and interagency working. This will result in the alignment of core competencies with measurable performance indicators that can be used by ICTs to establish goals and monitor their progress in relation to the achievement of those goals. Furthermore, guidelines for the maintenance of core competencies will be co-designed which will foster sustainable cultures of inter-professional collaboration among multidisciplinary teams working within the NICPOP.

The conclusion of this work package will result in the preparation of a final report by the research team members for the NCPOP. This report will present the work from the two co-design meetings in terms of an implementation framework. The report will highlight a working process model of core competencies for collaborative inter-professional working and provide a framework for achieving, maintaining and monitoring these competencies within multidisciplinary teams working in the ICPOP.

### Public and Patient Involvement

In line with the guidance from Involve UK ( http://www.invo.org.uk/) the involvement of public and patients has been incorporated into this project from the point of funding application and proposal development through to the dissemination of research outputs. Meaningful Public and Patient Involvement (PPI) in this project will not only ensure the quality and relevance of the research but also will ensure the research is informed by broader democratic principles and the values of accountability and transparency ( O’Donnell *et al.*, 2019). These values are fundamental to the principles of enabling IC. PPI is central to the success of the proposed ECLECTIC study.

Our PPI partner Age Friendly Ireland (represented by CM) has been involved in the development of ECLECTIC Funding Application. This organisation is focused on creating an inclusive, equitable society in which older people can live full, active, valued and healthy lives. Age Friendly Ireland has previously worked collaboratively with the Health Service Executive and the ICPOP by enabling the inclusion of the voice of older person in a partnership approach to identify challenges and co design solutions that are responsive to the needs of all older people. Together with co-author Ní Shé (the research lead in UCD for PPI, funding from the HRB PPI Ignite programme) we will nominate older people representing the two study sites. The nominated reps will be supported in their involvement via a capacity workshop in Work Package One. Following agreement of a terms of reference, nominated representatives will contribute to phases one and three and be part of the overall project steering group.

## Dissemination of results

The engagement of the NICPOP as the knowledge lead as well as a diverse group of co-applicants and collaborators is a deliberate strategy that will enable the development of highly relevant evidence. The NICPOP representation (JB, DO’S, HW, AC, PM, SQ, NM, NB and DL) on the project will enable the dissemination of ECLECTIC project outputs via an ongoing process of knowledge exchange activities in partnership with the NICPOP and the overall research team. This will ensure that the project, and its findings are far-reaching. This study proposal has been collaboratively developed in response to an identified need as outlined by the NICPOP.

This project will result in an implementation guide that will model core competencies for collaborative interprofessional working and provide a framework for achieving, maintaining and monitoring these competencies within multidisciplinary teams working in the NICPOP. The findings from this study will also be disseminated to the research community through the publications in international peer reviewed open access journals and presentations at national and international conferences and via the Co-Lead website (hosted by University College Dublin). Findings from the study will also be shared at community level through Age Friendly Ireland and its National Network of Older People’s Councils.

### Data availability

Anonymised and de-identified data underlying the results will be retained for five years and may be made available upon request as part of peer-reviewed article publications. Original audio recordings will be destroyed upon transcription.

## Study status

This study commenced in September 2019.

## Ethics approval and consent to participate

The research team has extensive experience in conducting research to a high ethical standard, and team members will ensure that ethical guidelines will be adhered to. The research team has received ethical approval for Work Package Two from the Midlands Research Ethics Committee (Ref: 040919DOD) and has received ethical exemption from the UCD Human Research Ethics Committee (LS-E-19-191-ODonnell). Access permissions and support for the study have been obtained from the CEO of CHO8 (Midlands), the Head of Service Social Care (HSE CHO9) and Head of Clinical Service (Beaumont). The research team will ensure that patient care is not compromised at any time due to staff participation in the research study. Informed written consent to participate in this study will be obtained from all participants from the two study sites. There is no use of randomisation, tissue samples or any invasive treatment in this study.

## Discussion

A policy of integration of health and social care along a continuum of care from the acute to the community is currently being adopted in Ireland and underpins the work of the NICPOP ( Ní Shé *et al.*, 2018b). Successful implementation of policy recommendation requires inter-professional collaborative approaches to care as well as a culture of collective leadership within multidisciplinary teams.

There is a recognised knowledge gap concerning how different HSCPs can work collaboratively together as part of multidisciplinary ICTs. Against this backdrop, the knowledge output from the ECLECTIC study fills a clear and stated need as identified by the NICPOP and the *SláinteCare* strategy ( SláinteCare, 2017).

Careful attention has been paid to address this need in the ECLECTIC study design which will result in the development of an implementation guide detailing the core competencies for collaborative inter-professional working. This will provide a framework for achieving, maintaining and monitoring these competencies within ICTs working in the ICPOP.

This project will deliver important and immediately applicable guidance for the NICPOP to enable them to implement the ECLECTIC project outputs nationally. The immediate beneficiary of this project are older people who will be better supported to live well at home in their communities by ICTs who are working collaboratively along a continuum of care.

The research will also contribute to the wider academic literature on collective leadership and IC. Learning from this project may also be applicable to other health care contexts nationally, achieving the goals for a new model of coordinated health and social care, shifting the focus of service delivery from acute to community as outlined in the *SláinteCare* strategy ( SláinteCare, 2017).

## Data availability

No data is associated with this article.
