# Supplementary material for: Embedding collective leadership to foster collaborative inter-professional working in the care of older people (ECLECTIC): Study protocol
Source: HRB Open Res. 2020 Mar 3;3:8. [Version 1] doi: 10.12688/hrbopenres.13004.1 (PMC7359747; doi:10.12688/hrbopenres.13004.1)
Supplement: Peer review history

# Peer review: Reviewer response for version 1

**DOI:** 10.21956/hrbopenres.14094.r27557

**Published:** 2020-07-13

**Authors:** Katie Robinson

**Affiliations:** 1 Department of Clinical Therapies, University of Limerick, Limerick, Ireland

Congratulations to the project team on a creative and well-theorized study with PPI involvement at all stages. The study is very well contextualized in Irish health policy and service delivery context.

 The following are suggested minor revisions: 

- Please include (pg 3) the date range over which the 13 IC teams were established to date.
- Clarify if medical professionals included in your working definition of ICT’s? (pg 3).
- The background section is detailed and the flow might be improved for the reader if the ECLECTIC project was briefly introduced at the start of paragraph # 5 (page 3) (paragraph opens with European Competency Framework). I think if the aim of ECLECTIC was reproduced here from the abstract that would help the reader.
- The authors present a convincing rationale for how collective leadership could foster interprofessional collaboration – it might be useful to very briefly summarize the limited literature on fostering IP collaboration including what is known about the impact of interprofessional pre-registration education and reference to the 2009 Cochrane review on practice based interventions for IP collaboration. This Cochrane review recommends that, alongside future RCTs, future qualitative exploration of how interventions affect collaboration and how improved collaboration contributes to changes in outcomes is warranted.  
- The ‘the co-design process’ is mentioned at the end of paragraph 4 page 4 – it is not fully clear at this stage what design process this refers to.
- The work-packages are presented with sufficient detail on design, data and analysis but could be restructured a little. I think it might be useful if all 3 WP’s opened with the objective they are addressing (from page 4) .
- WP2: Please clarify the focus of the initial qualitative interviews. No need to include an interview schedule but please describe what topics will be covered in the interviews and who will conduct them. You refer to ‘how team members interpret and understand IP collaboration in relation to their own disciplinary competencies’ in that paragraph and this is possibly the focus of the interviews? 
- The recruitment of individuals within the IC teams could be explicated further and how the consent process will be managed. It is not clear how HCP’s who do not wish to participate will be protected in the process.
- The approach to analysis of the qualitative interviews and observations could be described in a bit more detail.
- Some brief description of the composition of the teams included in the study and how long established they are would be useful.

Is the study design appropriate for the research question?

Yes

Is the rationale for, and objectives of, the study clearly described?

Yes

Are sufficient details of the methods provided to allow replication by others?

Yes

Are the datasets clearly presented in a useable and accessible format?

Not applicable

Reviewer Expertise:

NA

I confirm that I have read this submission and believe that I have an appropriate level of expertise to confirm that it is of an acceptable scientific standard.

# Peer review: Reviewer response for version 1

**DOI:** 10.21956/hrbopenres.14094.r27212

**Published:** 2020-03-30

**Authors:** Anne Hendry, Viktoria Stein

**Affiliations:** 1 International Centre for Integrated Care, University of the West of Scotland, Paisley, UK; 2 School of Social Policy, University of Birmingham, Birmingham, UK

The study protocol addresses an important challenge in the context of an ageing population in Ireland. It applies co-design principles and involves knowledge users and public and patient representatives to identify and support the core competencies for inter-professional collaborative working in health and social care teams providing services to older people. However the protocol does not confirm participation by informal carers. Nor is it clear that both PPR and carers will be involved in the educational sessions to ensure the lens of co-design is maintained throughout the study.

 The 2016 European Core Competences Framework is cited as the basis for developing these competencies. Surprisingly, there is no reference to more recent capability frameworks for older people that acknowledge the varying skill mix and capability within integrated teams and the role of the patient and carer as partners ^1^^,^^2^. 

 If the key aim of the study is to understand the competencies and cultures that underpin collaborative approaches to care, it may be more helpful to focus on the core competencies for integrated care as described by WHO ^3^, on the leadership skills required in complex systems ^4^, and to undertake comprehensive geriatric assessment and care planning ^5^. This would help to shift the focus from technical skills towards the relational skills required for integrated working and to forge strong, authentic relationships across the community, including with volunteers and community partner organisations ^6^: trust, resilience, managing uncertainty and complexity, curiosity, creativity and adaptability, willingness to acknowledge and explore differences and make better use of different skills and strengths, and an ability to surface and resolve conflicts.

 The working definition of ICT used is extremely broad and is more in keeping with a local clinical network for older people rather than an inter-professional team managing a defined caseload. There are some references to integrated intermediate care but also to a team-based case management approach to coordinating care of older people across the continuum of care. The scope and function of the 2 integrated teams participating in the study is not clear: do they provide time limited intermediate care, as defined in a recent international Delphi study ^7^; chronic case management, care and support; or a blend of both? Please clarify as this will have implications for the skill mix and competencies required by professionals, and for the approach to evaluation of the intervention. 

 Appropriate tools to assess inter-professional collaborative practice in a defined integrated team ^8^ differ from those to assess the maturity of collaboration within a wider clinical network. ^9^ A crucial factor, which has not been adequately addressed, is the question of how long the participating teams have been in place and the extent to which members have had an ongoing relationship. This will significantly influence their team performance and must be fully captured.

 Suggest there should also be a strong emphasis on structured assessment of the contextual factors surrounding the educational intervention, including finance, workforce capacity, skill mix, IT and leadership and management support, as workforce education and change in competencies are only one element of the complex intervention that is integrated care and are highly dependent on the delivery context ^10^.

 The co-design approach is welcome but it is not clear if the full range of professionals from both study sites will participate in the co-design workshops in the first phase. It would be counter intuitive to omit them as they are best placed people to determine their education and workforce development needs. It seems premature to pre-determine the topic and contents of the six Co-Lead modules that will meet these development needs. The researchers should approach this co-design workshop with appreciative inquiry principles so that selection of the most appropriate modules for the local context is an output from Work Package 1. 

 It is important to allow scope for picking up new themes which may emerge during all of the workshops. The protocol is ambitious in expectations from just 3 exploratory sessions. There is no discussion of how the researchers will deal with time lag and how they will adapt the schedule if the predetermined outcomes are not met, or if additional workshops are needed. Suggest add a section on study limitations and risks and mitigating actions.

 The protocol states that teams are to be observed as they undertake each educational module – how many professionals will be observed, by whom and how? How will the analysis of the observations be conducted? It is well established in the literature on qualitative research that the process of observation will influence the performance of the observed individuals towards what they think is expected of them. The methodology needs to discuss different biases, and how they will be mitigated.

 After completion of the training, it would be useful to add a further workshop or community forum to disseminate the learning and seek wider feedback in both areas to ensure relevance to both professionals and partners. The PPR participants are likely to be more articulate and more active than the average older person or carer receiving care and support services. Involving PPR is a great first step but there is a need to amplify this engagement by connecting and send checking with a wider group of people with lived experience. This will ensure a more inclusive community perspective and allow further refinement of the competencies and educational framework before progressing to Work Package 3.

 What is the time interval between completing the 6 Co-Lead modules and the final assessment to evaluate the efficacy of the intervention? It will be difficult to draw conclusions on sustainability of the Co-Lead intervention if the final assessment is conducted immediately after completing the training.

 It is not clear if the performance indicators to be developed will include validated assessments of inter-professional collaboration of the team (or network) as well as indicators of competencies achieved by individual professionals. Suggest such validated team assessment tools will be required as well as analysis of individual scores and of the observed discussion of the 2 scenarios. 

 Finally, a small but important point for those unfamiliar with the programme acronyms. NICPOP is variably described as the National Integrated Care Programme for Older People or Older Persons – the authors should be consistent. Should the reference to NCPOP in the fifth paragraph on page 7 be NICPOP? At one point, ICPOP is mentioned as well. Should that also be NICPOP?

Is the study design appropriate for the research question?

Yes

Is the rationale for, and objectives of, the study clearly described?

Yes

Are sufficient details of the methods provided to allow replication by others?

Partly

Are the datasets clearly presented in a useable and accessible format?

Not applicable

Reviewer Expertise:

Integrated care; ageing; frailty; inter-professional practice; intermediate care; inter-disciplinary team building; education and training for integrated care.

We confirm that we have read this submission and believe that we have an appropriate level of expertise to confirm that it is of an acceptable scientific standard.

## References

[1] : Frailty. A framework for core capabilities. *Health Education England, NHS England, Skills for Health*.2018;

[2] : European Collaborative and Interprofessional Capability Framework for Prevention and Management of Frailty-a consensus process supported by the Joint Action for Frailty Prevention (ADVANTAGE) and the European Geriatric Medicine Society (EuGMS). *Aging Clin Exp Res*.2020; 10.1007/s40520-019-01455-5 10.1007/s40520-019-01455-5 31970670PMC7170812

[3] : Competent health workforce for the provision of coordinated/ integrated health services. *Copenhagen: WHO Regional Office for Europe*.2015; Reference source

[4] : Leadership in integrated care systems.2018; Reference source

[5] : Understanding and improving multidisciplinary team working in geriatric medicine. *Age and Ageing*.2019;48(4) : 10.1093/ageing/afz021 498-505 10.1093/ageing/afz021 30855656

[6] : The Social Leadership Handbook (2nd Edition). *Sea Salt Publishing*.2016;

[7] : Intermediate care interventions for older adults: ADVANTAGE Joint Action for Frailty Prevention.2019;

[8] : Development and validation of the primary care team dynamics survey. *Health Serv Res*.2015;50(3) : 10.1111/1475-6773.12257 897-921 10.1111/1475-6773.12257 25423886PMC4450936

[9] : Effective networks for improvement: Developing and managing effective networks to support quality improvement in healthcare.2014; Reference source

[10] : Barriers and Facilitators to Workforce Changes in Integrated Care. *Int J Integr Care*.2018;18(2) : 10.5334/ijic.3587 17 10.5334/ijic.3587 30127701PMC6095071
